# 微小结节肺癌手术治疗方法综述及探讨

**DOI:** 10.3779/j.issn.1009-3419.2014.07.05

**Published:** 2014-07-20

**Authors:** 清泉 罗

**Affiliations:** 200030 上海，上海交通大学附属上海市胸科医院 Shanghai Chest Hospital Affiliated to Shanghai Jiao Tong University, Shanghai 200030, China

## 1 微小结节肺癌手术治疗的历史演变和意义

早至20世纪30年代，手术治疗肺癌的首选术式是全肺切除术，而同期为了治疗支气管扩张症等良性肺部疾病而施行的术式中已经出现了肺段切除术^[1, 2]^。随着外科技术的进步，遵循肿瘤手术的二大原则，即最大限度地切除肿瘤组织，最大限度地保留正常组织，从20世纪60年代开始，肺叶切除加淋巴结清扫术式逐渐发展成为胸外科医生治疗肺癌的标准术式^[3]^。在1995年，肺癌研究组（Lung Cancer Study Group, LCSG）^[4]^曾尝试将亚肺叶切除术（肺段切除术和楔形切除术）应用到肺癌的外科治疗中，进行了一项前瞻性随机临床实验的研究，全部入组的247例周围型临床分期T1N0M0的肺癌患者被随机分为亚肺叶切除组和肺叶切除组。亚肺叶切除术组的局部复发率明显高于肺叶切除组（12% *vs* 8%, *P*=0.008），虽然肺叶切除组的5年生存率高于亚肺叶切除组，但二者之间差异并无统计学意义（73% *vs* 56%, *P*=0.06）。因此，肺叶切除术作为外科标准术式在肺癌治疗中的地位再次得到了巩固。

近年来，螺旋计算机断层扫描（computed tomography, CT）的影像技术的发展提高了肺部小结节，尤其是非实性结节的检出率，并由此产生大批早期肺癌高危人群。相较于1995年LCSG的试验组，这批新的群体的肺部结节更小，更隐匿。加上高龄和心肺功能受限患者群体的增加，促使我们重新审视判断亚肺叶切除治疗早期非小细胞肺癌的有效性。

1997年，Kodama等^[5]^报道了一项临床分期T1N0非小细胞肺癌的病例对照研究，该研究以治愈为目的，有计划实施亚肺叶切除术。所有46例研究对象均为肿瘤≤2 cm的周围型肺癌患者，接受肺段切除加淋巴结清扫。对照组77例Ⅰ期患者平均肿瘤大小22.9 mm接受肺叶切除。结果肺段切除组5年生存率93%，和肺叶切除组相似。局部复发率高于肺叶切除组（2.2% *vs* 1.3%）。

2001年，Okada^[6]^也分析了70例T1N0（肿瘤≤2 cm）非小细胞肺癌患者接受扩大肺段切除术和对照组139例肺叶切除术的对照研究，所谓的扩大性肺段切除术就是切除范围比解剖性肺段切除多一点肺实质。这项研究中肺段切除只在肺段、肺门、纵隔淋巴结术中冰冻检查无转移的情况下进行，一旦发现淋巴结转移则进行肺叶切除。对于病理分期T1N0的患者，扩大肺段切除组5年生存率为87.1%，肺叶组5年生存率为87.7%（*P*=0.8）。扩大肺段切除组没有局部复发肺叶切除组的局部复发率没有报道。

2006年，一项日本多中心非随机前瞻性研究^[7]^，对亚肺叶切除（扩大肺段切除305例）与肺叶切除（262例）进行比较。两者之间无病生存期（disease-free survival, DFS）和生存期（overall survival, OS）无差异，亚肺叶切除组的5年DFS和OS分别为85.9%和89.6%，肺叶切除组的5年DFS和OS分别为83.4%和89.1%。复发率方面，亚肺叶组为4.9%，肺叶组为6.9%。结论是，对T1N0、肿瘤≤2 cm的非小细胞肺癌，亚肺叶切除是一种可以替代肺叶切除的术式。

鉴于以上不同手术组争论焦点，全球范围内正进行二项大型多中心临床试验：

一项是Ⅲ期多中心随机对照研究亚肺叶切除和肺叶切除治疗周围型非小细胞肺癌（≤2 cm）的临床试验，由美国国家癌症研究所（National Cancer Institute, NCI）牵头，美国和加拿大149个研究组参与。研究对象为经CT扫描怀疑或证实肺部肿瘤≤ 2 cm、位于肺外周三分之一的周围型肺癌且淋巴结为阴性的患者，纯GGO除外。术中冰冻切片病理检查证实非小细胞肺癌的诊断，且为N0状态（右侧4、7、10组淋巴结阴性，左侧5、6、7、10组淋巴结阴性）。随机分为肺叶切除组和亚肺叶切除组二组，从2007年6月起，计划5年入组1, 258例患者。试验的主要研究终点是无病生存期，次要终点是总生存期、局部或全身复发转移率以及术后6个月呼气流量测定的肺功能。术后随访每6个月一次×2年，然后改为每年一次×5年，计划至2021年3月完成出结果。

另一项是由日本临床肿瘤组（Japan Clinical Oncology Group）和西日本肿瘤组（West Japan Oncology Group）联合研究，全日本71个研究组参与的Ⅲ期多中心随机对照研究亚肺叶切除和肺叶切除治疗周围型非小细胞肺癌的临床试验。研究对象为经CT扫描怀疑非小细胞肺癌为单发的、位于肺外周三分之一的周围型肺癌、肿瘤最大直径≤2 cm且淋巴结为阴性的患者，影像学诊断非侵袭性癌除外。随机分为肺叶切除组（550例）和亚肺叶切除组（肺段切除）（550例）对照。从2009年8月起，计划3年入组1, 100例患者。试验的主要研究终点是总生存期，次要终点是术后肺功能、无病生存期、局部复发率、不良事件、完成肺段切除患者的比例、住院时间、胸管引流时间、手术时间、失血量以及切割闭合器使用数量。术后随访至少5年，先每6个月一次×2年，然后改为每12个月一次。

相信结果揭晓时，可以有一个最客观的定论来规范微小结节肺癌外科治疗的标准术式。

## 2 微小结节肺癌手术的适应症（时间、合适的人群）

肺部微小结节在临床上越来越常见，并且对于临床医生而言是个棘手的问题。根据美国胸科医师协会（American College of Chest Physicians, ACCP）第三版临床指南，外科手术用于大于8 mm的实质性结节主要是基于以下几个方面：①临床的恶性概率很高（＞65%）；②正电子发射计算机断层显像（positron emission tomography, PET）提示结节代谢增高或者其他功能影像提示强阳性；③非手术的活检结果怀疑为恶性；④患者需要进行明确诊断。而手术方法主要推荐是微创胸腔镜手术，肺叶切除或亚肺叶切除。当切除小的或者深的结节时，可能要考虑进行高级的定位方式或者开胸切除术^[8]^。

当实质性结节直径小于8 mm时，如前所述，这种结节的恶性程度相对较低，若患者未提示具有肺癌的发病风险，建议患者行非增强的低剂量CT扫描监测，具体频率和间隔时间应根据结节大小而定^[9-13]^。

而另一类性质的结节，即非实质性结节，分为纯磨玻璃样影和部分磨玻璃样部分实质结节。CT提示纯磨玻璃样影，若直径小于5 mm，则不需进行进一步的检查评估。当直径大于5 mm时，应选择每年进行CT扫描监测至少3年。部分实质（大于50%的磨玻璃样）结节直径小于8 mm时，应选择非增强的薄层CT扫描，在3、12、24个月时进行扫描，后续每年进行一次CT扫描，至少1年-3年。对非实质性结节而言，CT扫描应选择非增强的薄层扫描。当非实质性结节增长或发展至实质结节，通常提示恶性可能，应考虑进行进一步的评估检查或手术切除。直径大于10 mm的非实质性结节，应进行3个月的早期随访，若结节持续存在，考虑后续性非手术性活检或者手术诊断^[9, 14, 15]^。

部分实质结节直径大于10 mm时，建议前3个月重复CT扫描，若结节持续存在，进行PET检查、非手术性的活检和/或手术切除的后续评估手段。当部分实质结节内部的实质部分直径小于8 mm时，不推荐行PET分析病变性质。而直径大于15 mm的部分实质结节则直接进行后续的PET检查、非手术性的活检和/或手术诊断等检查^[16, 17]^。除以上所述结节分类，若患者存在一个主要的结节及一个以上的其他小结节，除非有组织病理确诊为转移，否则应每个结节都单独分析评估，而且后续治疗不能拖延。这种具有一个以上肺结节的肺癌患者的分类和合适治疗方案很难确定，需要进行多学科综合分析^[9, 18]^。

## 3 微小结节肺癌手术的方法

由于影像技术的发展，我们诊断发现微小结节肺癌的能力也获得了革命性的进步。与之同步，外科手术技术的发展也愈发广泛应用于更多患者群体，甚至包括一些心肺功能受限人群。微创胸腔镜（video-assisted thoracic surgery, VATS）技术，虽然没有纳入LCSG的以往临床实验，但自20世纪90年代始，便日益广泛应用于胸外科手术，至今已经形成了一个全新的微创胸外科时代。

VATS技术治疗微小结节肺癌（≤2 cm肿瘤）的远期疗效已经被证实等同于开放手术，而围术期优势包括更少的失血量、更快的术后恢复、更多的肺功能保存和更短的住院时间^[19-21]^。

由于技术上的简便和相对较少的围术期并发症死亡率，VATS楔形切除经常被用于亚肺叶切除治疗微小结节肺癌。另一方面，高龄患者、合并心肺功能不全患者手术常常要求较短的手术时间和较轻的伤口疼痛，也是促使外科医生选择此类手术的重要原因^[22-24]^。

Mayo Clinic的Miller等^[25]^专门对≤1 cm肿瘤施行肺叶切除、亚肺叶切除（肺段切除和楔形切除）进行对比。尽管肺叶切除组有明显的生存优势，但在亚肺叶切除的亚组分析中，无论是生存率优势还是局部复发率控制优势，都没有发现存在统计学差异。

Okada等^[26]^和El-Sherif等^[27]^在大型回顾性研究中均报道了影响局部复发的关键因素，指出楔形切除后具有较高的局部复发的原因主要是技术上的局限，包括切缘阳性、肺实质内和肺门淋巴结的不完全切除。

相较于VATS楔形切除，VATS解剖性肺段切除具有的生存优势和较少的局部复发率主要和以下几个技术特点密切相关：①严格按解剖面进行切除；②切除范围足够大；③肺实质内引流淋巴组织的切除。肺癌术后发生局部复发的首要原因包括肺叶内肿瘤残留、肺叶内淋巴结转移和切缘阳性^[28, 29]^。VATS解剖性肺段切除较VATS楔形切除更接近肿瘤手术切除要求，更好地解决了肿瘤局部复发的难题。但需要指出的是，无论选择何种亚肺叶切除，建议术中评估淋巴结状态，以取得更全面的肿瘤分期，以便和肺叶切除疗效有更完整对比^[30-33]^。

有一种观点认为施行亚肺叶肺段切除的肿瘤一定要局限于肺段的解剖界限内，而不应跨段。尽管肺段切除可以应用于中央型肺癌，但是大量实践证明具有治愈性的肺段切除术应该应用于位于肺外周三分之一的周围型非小细胞肺癌。这主要是因为中央型肺癌往往包含淋巴结转移，预后较差。Ketchedjian等^[34]^报道T1中央型肺癌淋巴结转移检出率达50%。Lee等^[35]^报道中央型肺癌发生N2淋巴结转移，在肿瘤＞2 cm中高达26.7%，与之形成鲜明对比的是，在周围型肺癌中，这一比例仅为2.9%。

中央型肺癌较高的淋巴结转移率表明肿瘤位于较丰富淋巴引流区域时，表现出的更广泛的侵袭性，此时强调肺叶切除术的效果毫无疑问要优于亚肺叶切除。

另外值得注意的是，Sienel等^[36]^研究结果发现S1-S3肺段切除术后复发率明显高于S4-S10肺段切除（23% *vs* 5%），但没有解释该现象。

因此，我们强调术中冰冻病理检查区域淋巴结状态，明确有否局部淋巴结转移，能有效帮助外科医生正确判断选择手术方式，提高手术疗效。

基于LCSG的报道，肺叶切除已然成为外科治疗非小细胞肺癌的标准术式。肺叶切除加淋巴结清扫能使肺癌患者得到最大的生存获益，亚肺叶切除主要适合心肺功能受限的患者。然而，最近证据表明，特别是大型荟萃分析结果支持对于肿瘤≤2 cm、周围型Ⅰ期微小结节肺癌患者选择性实施亚肺叶切除，尤其推荐解剖性肺段切除。这种手术方式安全，远期生存率等同于肺叶切除术。关键在于术中冰冻病理检查评估淋巴结状态以除外淋巴结转移，完善准确的疾病分期，以正确判断决定合适的手术方式。

## 4 胸科医院手术的微小结节肺癌分析

上海市胸科医院肺部肿瘤临床医学中心外科组自2008年1月-2012年12月施行胸腔镜治疗785例拟诊微小结节肺癌患者，病理信息如下：

### 1.1 腺癌按大小分类

①肿瘤大小＞0.8 cm共453例，其中原位腺癌25例、微浸润性腺癌29例、浸润性腺癌399例；②0.5 cm＜肿瘤大小≤0.8 cm共22例，其中原位腺癌12例、微浸润性腺癌5例、浸润性腺癌5例；③肿瘤大小≤0.5 cm共6例，其中原位腺癌3例、微浸润性腺癌2例、浸润性腺癌1例。

### 1.2 良恶性按大小分类

①肿瘤大小＞0.8 cm共618例，其中良性肿瘤116例，恶性肿瘤/癌502例；②0.5 cm＜肿瘤大小≤0.8 cm共33例，其中良性肿瘤10例，恶性肿瘤/癌23例；③肿瘤大小≤0.5 cm共10例，其中良性肿瘤4例，恶性肿瘤/癌6例。

### 1.3 淋巴结转移与腺癌类型的关系

①原位腺癌40例中，均未发生淋巴结癌转移；②浸润性腺癌共423例，71例发生淋巴结癌转移，其中70例肿瘤直径大小＞0.8 cm；③微浸润性腺癌37例中，共2例发生淋巴结癌转移，其肿瘤大小＞0.8 cm。

我们的经验是结节直径大于0.8 cm，结合患者情况，推荐手术治疗；病理类型是原位腺癌的可仅行淋巴结采样；手术方法推荐微创胸腔镜手术。
